# MiR-375 reduces the stemness of gastric cancer cells through triggering ferroptosis

**DOI:** 10.1186/s13287-021-02394-7

**Published:** 2021-06-05

**Authors:** Haiwei Ni, Hai Qin, Cheng Sun, Yichen Liu, Guojing Ruan, Qianqian Guo, Tao Xi, Yingying Xing, Lufeng Zheng

**Affiliations:** 1 School of Life Science and Technology, Jiangsu Key Laboratory of Carcinogenesis and Intervention, China Pharmaceutical University, 639 Longmian Road, Nanjing, 211198 People’s Republic of China; 2 The Affiliated Hospital of Nanjing University of Chinese Medicine, Nanjing, Jiangsu 210029 People’s Republic of China; 3 Department of Pharmacy, the Affiliated Cancer Hospital of Zhengzhou University, Henan Cancer Hospital, Zhengzhou, Henan People’s Republic of China

**Keywords:** MiR-375, SLC7A11, Ferroptosis, Stemness, Gastric cancer

## Abstract

**Background:**

Gastric cancer stem cells (CSCs) are the main causes of metastasis and drug resistance. We previously indicated that miR-375 can inhibit *Helicobacter pylori*-induced gastric carcinogenesis; here, we aim to explore the effects and mechanisms of miR-375 on gastric cancer (GC) cell stemness.

**Methods:**

Lentivirus infection was used to construct GC cells with ectopic expression of miR-375. In vitro and in vivo experiments, including analysis of tumor spheroid formation, CD44+ sub-population with stemness, stemness marker expression, and tumor-initiating ability, were performed to evaluate the effects of miR-375 on the stemness of GC cells. Furthermore, microarray and bioinformatics analysis were performed to search the potential targets of miR-375 in GC cells. Luciferase reporter, RNA immunoprecipitation, and RNA-FISH assays were carried out to verify the targeting of miR-375. Subsequently, combined with tissue microarray analysis, erastin-resistant GC cells, transmission electron microscopy, a series of agonists and oxidative stress markers, the underlying mechanisms contributing to miR-375-mediated effects were explored.

**Results:**

MiR-375 reduced the stemness of GC cells in vitro and in vivo*.* Mechanistically, SLC7A11 was identified as a direct target of miR-375 and miR-375 attenuated the stemness of GC cells mainly through triggering SLC7A11-dependent ferroptosis.

**Conclusion:**

MiR-375 can trigger the ferroptosis through targeting SLC7A11, which is essential for miR-375-mediated inhibition on GC cell stemness. These results suggest that the miR-375/SLC7A11 regulatory axis could serve as a potential target to provoke the ferroptosis and thus attenuate the stemness of GC cells.

**Supplementary Information:**

The online version contains supplementary material available at 10.1186/s13287-021-02394-7.

## Introduction

Gastric cancer (GC) ranks fifth in incidence among all malignant tumors worldwide and the mortality rate is third (powered by GLOBOCAN 2018); this is closely due to tumor relapse, metastasis, and drug resistance. Cancer stem cells (CSCs) have been regarded as the root of tumor relapse, metastasis, and drug resistance [1, 2]. As a consequence, it may facilitate GC treatment to elucidate the underlying mechanisms contributing to the progression gastric CSCs.

Ferroptosis, a newly established form of cell death, is different from conventional programmed death such as apoptosis, necrosis, and autophagy [3]. Biochemically, ferroptosis is initiated by oxidative perturbations of intracellular microenvironment which involves lipid peroxidation, lethal reactive oxygen species (ROS), and free ferrous iron (Fe^2+^) [4]. Although cancer cells usually have a higher level of ROS, which is essential for tumor progression, such as migration, proliferation, and survival [5, 6], while CSCs are believed to possess a lower level of ROS [7]. Thus, it might be effective to eliminate CSCs by stimulating ROS generation [8]. Simultaneously, cancer cells can be eliminated via elevating ROS with inhibiting antioxidant defense [9]. Therefore, it encourages us to focus on a ROS-dependent cell death mechanism for gastric CSCs. Notably, iron metabolism [10, 11] and ferroptosis have recently been shown as a promising therapeutic strategy to eliminate CSCs [12–14]. Meanwhile, ferroptosis is associated with chemoresistance and the anti-tumor efficacy of immunotherapy [15, 16]. However, the involvement of ferroptosis in gastric CSC progression was still confusing.

A number of genes required for ferroptosis have been identified, including those involved in lipid peroxidation and amino acid metabolism, in which system xc-, a sodium-independent antiporter of cystine and glutamate, takes part in amino acid metabolism [3]. Cystine-glutamate antiporter xCT (SLC7A11), which is the subunit of system xc-, together with SLC3A2 (CD98), is selective for uptaking cystine into cells in exchange for glutamate [17]. This system synthesizes glutathione (GSH) by taking extracellular cystine into cells and thus represses oxidative stress response during ferroptosis. Many studies have found that xCT is abnormally expressed due to the overloaded oxidative stress and increased nutrient metabolism requirements in various carcinomas [18]. CD44, which is the cell adhesion molecule, has been identified as a marker of gastric CSCs [19, 20]. The previous studies have shown that a CD44 variant (CD44v) interacts with xCT [21, 22] and SLC7A11 overexpression is associated with increased CSC-like properties [23]. However, the relationship between SLC7A11 and the stemness in GC is still undefined.

MicroRNAs are short endogenous and evolutionarily conserved single-strand RNA molecules, approximately 19 to 25 nucleotides in size [24] and have been widely shown to be associated with CSC-like traits [25]. MiR-375 is discovered as a pluripotent miRNA involved in pancreatic islet development, glucose homeostasis, and cell differentiation [26]. Additionally, miR-375 has been identified as a tumor suppressor in various tumors [27, 28]. Notably, miR-375 is found to be lowly expressed and considered as a possible biomarker in GC [29, 30]. We previously showed that miR-375 can inhibit *Helicobacter pylori*-induced gastric carcinogenesis [31]. Furthermore, ours and other studies have indicated that miR-375 can suppress the stemness of breast cancer cells [32]. Thus, we assume that miR-375 may play critical roles in GC cell stemness.

Here, we found that miR-375 suppressed the stemness of GC cells in vitro and in vivo. Further mechanistic studies revealed that miR-375 could induce ferroptosis by targeting SLC7A11 and thus attenuate the stemness of GC cells. This study identified a novel miR-375/SLC7A11 regulatory axis responsible for GC cell stemness by mediating ferroptosis. Since conventional chemotherapeutic drugs which induce cancer cell apoptosis have been confirmed to induce cancer cell stemness and drug resistance, this miR-375/SLC7A11 regulatory axis might serve as a potential target to suppress the stemness of GC cells via triggering ferroptosis and provide clues for the development of ferroptosis inducer.

## Materials and methods

### Cell culture and chemical agents

Human GC cell lines SGC-7901 and BGC-823 were obtained from China Academia Sinica Cell Repository (Shanghai, China). Human gastric mucosal epithelial cell line GES-1 was a gift from the Shanghai Institute of Digestive Disease. Cells were cultured in RPMI-1640 medium (KeyGEN BioTECH, Nanjing, China) supplemented with 10% fetal bovine serum (FBS, Gibco, Grand Island, NY, USA), 100 μg*/*mL streptomycin, and 100 units*/*mL penicillin [33, 34]. HEK-293 T cells preserved in our laboratory were cultured in high glucose DMEM (KeyGEN BioTECH, China). All cell lines were incubated at 37 °C in 5% CO_2_. Ferroptosis agonists erastin, sorafenib, and ferroptosis inhibitor Ferrostatin-1 (Fer-1) were purchased from MCE (MedChemexpress, USA). Another ferroptosis agonist sulfasalazine (SAS), pan-caspase inhibitor Z-VAD-FMK, and necrosis inhibitor Necrostatin-1 (Nec-1) were purchased from TargetMol (Target Molecule Corp, USA).

### Plasmids, miR-375 mimics, and siRNA transfection

When cell growth reached 40–50% in density, cells were used for transfection using jetPRIME transfection reagent (Polyplus Transfection, France) following the manufacturer’s protocol. MiR-375 mimics and miRNA NC (negative control) were synthesized by GenePharma (Shanghai, China). Small interfering RNA (siRNA) of SLC7A11 (5′-GGAGTTATGCAGCTAATTA-3′; 5′-GGGTGGAACTCCTCATAAT-3′; 5′-GGGAACAACTATAAAGAAA-3′) or si-NC (5′-TTCTCCGAACGTGTCACGT-3′) were synthesized by Biomics Biotechnology (Nantong, China). SLC7A11 overexpression vector pcDNA3.4-SLC7A11 and empty plasmid (NC) were purchased from MiaolingBio (Wuhan, China). For luciferase reporter assay, the sequences of SLC7A11 3′UTR (WT) containing miR-375 binding site and SLC7A11 3′UTR (MUT) containing mutant miR-375 binding site were cloned into pMIR-Report, respectively. The detailed procedure for plasmid construction was referred in our previous work [35]. All plasmids were verified by DNA-sequencing.

### Quantitative real-time PCR (qRT-PCR)

Total RNA was extracted from GC cell lines using TRIzol (TransGen Biotech, China) following the standard recommendation. The cDNA was reversely transcribed with M-MLV (Vazyme, Nanjing, China). MiRNA and U6 snRNA RT primer mix and miRNA qRT-PCR primer set (GenePharma, China) were used to detect and quantify miRNA expression. SYBR Green master mix (Vazyme) was used for qRT-PCR assay on an ABI Prism 7500 Sequence Detector (Applied Biosystems, USA). The primer sequences used were listed as below: SLC7A11 forward, 5′-TCTCCAAAGGAGGTTACCTGC-3′; SLC7A11 reverse, 5′-AGACTCCCCTCAGTAAAGTGAC-3′; GAPDH forward, 5′-TGTGGGCATCAATGGATTTGG-3′; GAPDH reverse, 5′-ACACCATGTATTCCGGGTCAAT-3′. The mRNA and miRNA levels were normalized to an internal control (GAPDH or U6 snRNA), respectively. The relative expression level was calculated using 2^−ΔΔCt^ method [36].

### Construction of erastin-resistant GC cells

Erastin-resistant cells were constructed as a ferroptosis-resistant model [16]. To generate erastin-resistant GC cells, BGC-823 or SGC-7901 cells were seeded in 24-well culture plates and treated with erastin (10 μM) for two weeks. Fresh culture medium containing erastin was changed every 2 days. Then, surviving clones were collected and further amplified. The resistance of erastin was confirmed by cell viability assay [37].

### Stable cell line construction with lentivirus infection

Briefly, miR-375, SLC7A11 coding, and SLC7A11 shRNA sequences were sub-cloned into pGLV3/H1/GFP+Puro Vector (LV3 lentiviral vector) (GenePharma), respectively. The lentivirus package was performed by GenePharma. The final viral titer was 1 × 10^8^ TU/mL and 20 μl lentivirus was added into 1 mL medium to infect GC cells. Stable cell lines were selected with puromycin (Sigma, 2 μg/mL) for 2 weeks. Fluorescence observation and qRT-PCR were used to verify infection efficiency [38].

### Tissue microarray analysis

The tissue microarray was purchased from OutdoBiotech (Shanghai, China), which contains 17 primary GC tissues, 12 normal adjacent tissues, 16 metastatic lesions, and 5 normal gastric mucosa tissues [39]. Further immunohistochemistry assay was performed by Servicebio (Wuhan, China) to detect SLC7A11 expression which was quantified using Quantity-one Software. Additionally, RNA-fluorescence in situ hybridization assay was performed by Boster Biological Technology to detect miR-375 and SLC7A11 levels in this tissue microarray.

### Microarray analysis

Total RNA was extracted in SCG7901 cells with or without miR-375 stable overexpression using Trizol reagent (Life Technologies, Carlsbad, CA, USA) and purified with an RNeasy mini kit (Qiagen, Valencia, CA, USA). Biotinylated cDNA was prepared according to the standard Affymetrix protocol from 250 ng total RNA using Ambion® WT Expression Kit. Following labeling, fragmented cDNA was hybridized for 16 h at 45 °C on Clariom ™ D Assay (Human, Affymetrix). GeneChips were washed and stained in the Affymetrix Fluidics Station 450. All arrays were scanned by using Affymetrix® GeneChip Command Console (AGCC) which was installed in GeneChip® Scanner 3000 7G [40]. The operation of microarray analysis was outsourced by Beijing Cnkingbio Biotechnology Corporation (Cnkingbio, China). All data was deposited in Gene Expression Omnibus (GEO) repository (GSE147698).

### Western blotting

The detailed procedure was described in our previous study [41]. Firstly, cells were scraped from the 6-well culture plates for centrifugal collection at 300×*g* for 5 min at 4 °C. Cell-derived protein lysate was harvested by RIPA lysate (Beyotime, China). Ten micrograms of protein was fractionated by 10% SDS/PAGE, transferred to polyvinylidene difluoride membranes, and then incubated with the primary antibody at 4 °C overnight. After incubating with a secondary antibody for 45 min, the ECL system was used to detect the intensity of protein expression [42, 43]. Primary antibodies were SLC7A11/xCT (1:1000, proteintech, China), ALDH1A1 (1:1000, proteintech, China) and Oct3/4 (1:1000, Wanlei, China), CD44 (1:1000, Wanlei, China), and GAPDH (1:5000, YI FEI XUE BIOTECHNOLOGY, Nanjing, China) was used as an internal reference.

### RNA immunoprecipitation (RIP) assay

RIP assay was performed using the Protein A/G Agarose Resin (YEASEN, China) following the manufacturer’s protocol. For RIP assay, SLC7A11 3′UTR sequences containing miR-375 binding sites or mutated binding sites were inserted into pcDNA3.1 (+) plasmid, named as SLC7A11 3′UTR (WT) or SLC7A11 3′UTR (MUT), respectively. SGC-7901 and BGC-823 cells were transfected with SLC7A11 3′UTR (WT) and SLC7A11 3′UTR (MUT). After 24 h, cells were extracted and lysed by NP-40 lysis buffer (Beyotime, China). After that, 100 μL lysate was incubated with NP-40 buffer containing Protein A/G Agarose Resin bound with human anti-Ago2 antibody (Cell Signaling Technology, Danvers, MA, USA) at 4 °C overnight. Agarose resin was isolated by centrifugation and digested with protease K (YEASEN) to isolate Ago2-RNA complex from the beads. qRT-PCR was performed to detect miR-375 level [44].

### RNA-fluorescence in situ hybridization (RNA-FISH)

RNA-FISH for miR-375 and SLC7A11 was performed on GC cells. Cell slides fixed with 4% paraformaldehyde were treated with several buffers in RNA-FISH Kit (GenePharma). After denaturing at 73 °C for 5 min, probe mixture was hybridized in cell slide overnight in darkness at 37 °C. 5′CY3-labeled Locked Nucleic Acid probes targeting miR-375 and 5′FAM labeled Locked Nucleic Acid probes targeting SLC7A11 (GenePharma). After washing, the signal was observed under confocal microscope. Detailed procedures were followed by the standard protocol (GenePharma) [44].

### Dual-luciferase reporter assay

MiR-375 mimics or miR-375 NC and SLC7A11 3′UTR (WT), SLC7A11 3′UTR (MUT), or pMIR-Report were co-transfected into HEK293T cells using JetPRIME. After 48 h, cell lysate was collected following the instructions’ protocol of Dual Luciferase Reporter Gene Assay Kit (Yeason, China). The luciferase activity was quantified by POLARstar Omega multimode microplate reader. The relative luciferase activity was calculated by firefly luciferase activity/Renilla luciferase activity [44].

### Flow cytometry analysis

BGC-823 and SGC-7901 cells were seeded into 6-well plates and transfected with miR-375 mimics, si-SLC7A11, pcDNA3.4-SLC7A11(SLC7A11), or pcDNA3.4 (NC). Flow cytometry analysis was performed to detect CD44+ sub-population [19]. Briefly, cells were harvested at a density of 10^6^ cells/mL by centrifugation at 300×*g* and 4 °C for 5 min. Cells were stained with anti-CD44-APC (BD Biosciences, USA) or an isotype control at 4 °C in darkness. The stained cells were washed using PBS and measured by flow cytometer (BD, USA) [43].

### Cell viability assay (Cell Counting Kit-8)

Cell viability was assessed using CCK-8 (Cell Counting Kit-8) assay (Target Mol, USA). Cells were seeded in a 96-well plate at a density of 3000 cells/well in 100 μL of culture medium in a 5% CO_2_ incubator at 37 °C for 24 h. Then, different concentrations of erastin, sorafenib, or sulfasalazine were added to the culture plate, and DMSO was used as a NC group. After incubating for 24 h, 10 μL CCK-8 was added to each well for 1 h incubation. The absorbance was measured at 450 nm with a microplate reader [45].

### Spheroid formation assay

GC cells were cultured in serum-free DMEM/F12 medium supplemented with B27 supplement, 20 ng/mL EGF, and 20 ng/mL bFGF (ThermoFisher Scientific, USA) in 96-well ultra-low attachment dishes (Corning, USA) [46]. Two hundred cells were seeded into 200 μL serum-free medium per well for at least 7 days [47]. Spheres with the size bigger than 70 μm were counted and obtained. All images were recorded with a Leica DMI microscope (DE).

### Nude mouse xenograft models and metastasis experiment

All in vivo experiments with mice xenotransplant model were performed in accordance with the approval of the Ethics Committee for Animal Experimentation of China Pharmaceutical University. And the experiments were complied with the ARRIVE guidelines and should be carried out in accordance with the UK Animals (Scientific Procedures) Act, 1986. Four- to eight-week-old female BALB/c nude mice were purchased from the Model Animal Research Center of Nanjing University (Nanjing, China). For the tumor-limiting dilution assay, 1 × 10^7^, 5 × 10^6^, and 2.5 × 10^6^ of LV3-miR-375 cells, LV3-SLC7A11 cells, LV3-shSLC7A11 cells, LV3-miR-375-SLC7A11 cells, and LV3-NC cells (BGC-823 and SGC-7901) were subcutaneously implanted into the underarm of mice. Fifteen days later, all mice were euthanized and tumor tissues were collected and weighed. The stem cell frequencies were calculated using an ELDA (http://bioinf.wehi.edu.au/software/elda/) [48]. For metastasis experiment, mouse models were established by intravenous injection of cells. Three mice per group were injected with 2 × 10^6^ cells in 200 μL RPMI-1640 serum-free media. Six weeks after injection, mice were sacrificed for collecting lung tissues. Tissue samples were then sectioned and stained with hematoxylin-eosin. All procedures and image scanning were conducted by Servicebio (Wuhan, China).

### Glutathione (GSH), intracellular cysteine, and lipid peroxidation assays

Total GSH levels in six-well cell plates were determined using a Glutathione Assay Kit (Beyotime, China) following the standard protocol. The intracellular cysteine test kits were purchased from Nanjing Jiancheng Bioengineering Institute (Nanjing, China). Cysteine level was measured using Cysteine (Cys) content test kit (A126-1-1) (Nanjing Jiancheng Bioengeering Institute, Nanjing, China) in the six-well cell plates according to the manufacturer’s protocol. C11-BODIPY^581/591^ (Thermo Fisher Scientific, Waltham, MA, USA) was used to detect the level of lipid peroxidation. Cells were incubated in RPMI-1640 medium with C11-BODIPY^581/591^(1 μM) for 30 min at 37 °C. Flow cytometry was carried out at the absorbance of 484/510 nm [3].

### Transmission electron microscopy

Cells were added with electron microscopy fixative at 4 °C for 24 h and centrifuged at low speed, then wrapped in 1% agarose and rinsed 3 times with 0.1M PBS (PH = 7.4) for 15 min each time. Then, cells were fixed with 1% osmic acid-0.1M PBS (PH = 7.4) at room temperature (20 °C) for 2 h. Subsequently, cells were rinsed with 0.1 M PBS (pH = 7.4) 3 times for 15 min each time. In the period of dehydration, the samples were dehydrated by placing 50%, 70%, 80%, 90%, 95%, 100%, and 100% alcohol and 100% acetone in order for 15 min each time, respectively. After being embed with 812 embedding agent for 5–8 h, the samples were inserted into the embedding plate for 37 °C over night. Next, the samples were polymerized in an oven at 60 °C for 48 h. Samples were cut into ultra-thin slices (60–80 nm) with ultra-thin microtome. After uranium-lead double staining, the sections were dried at room temperature overnight and observed under the transmission electron microscope [49].

### Prussian blue staining

Prussian blue staining kit was purchased from Servicebio (Wuhan, China). The paraffin embedded slices of GC tumor tissues were continuously transferred into xylene I for 20 min, xylene II for 20 min, anhydrous ethanol I for 5 min, anhydrous ethanol II for 5 min, and 75% alcohol for 5 min, respectively. Then, the slices were washed with distilled water. After mixing the Prussian blue dye A and B in equal proportions into the Prussian blue dye solution, the slices were put into the dye solution for 1 h and washed twice with distilled water. Nucleus staining was performed with Prussian blue C for 3 min and rinsed with running water. Subsequently, slices were continuously put into anhydrous ethanol I for 5 min, anhydrous ethanol II for 5 min, anhydrous ethanol III for 5 min, xylene I for 5 min, and xylene II for 5 min, respectively. Finally, the slides were sealed with neutral gum and observed under microscope [50].

### Statistical analysis

GraphPad Prism 5 (GraphPad Software, Inc., La Jolla, CA, USA) was utilized for the statistical analysis. All results were obtained from three independent experiments and presented as the mean ± standard deviation (SD). The statistical significance between different groups was analyzed by Student’s *t* test. P < 0.05 or less was considered to be statistically significant.

## Results

### MiR-375 inhibits the stemness of GC cells in vivo and in vitro

**Fig. 1:**
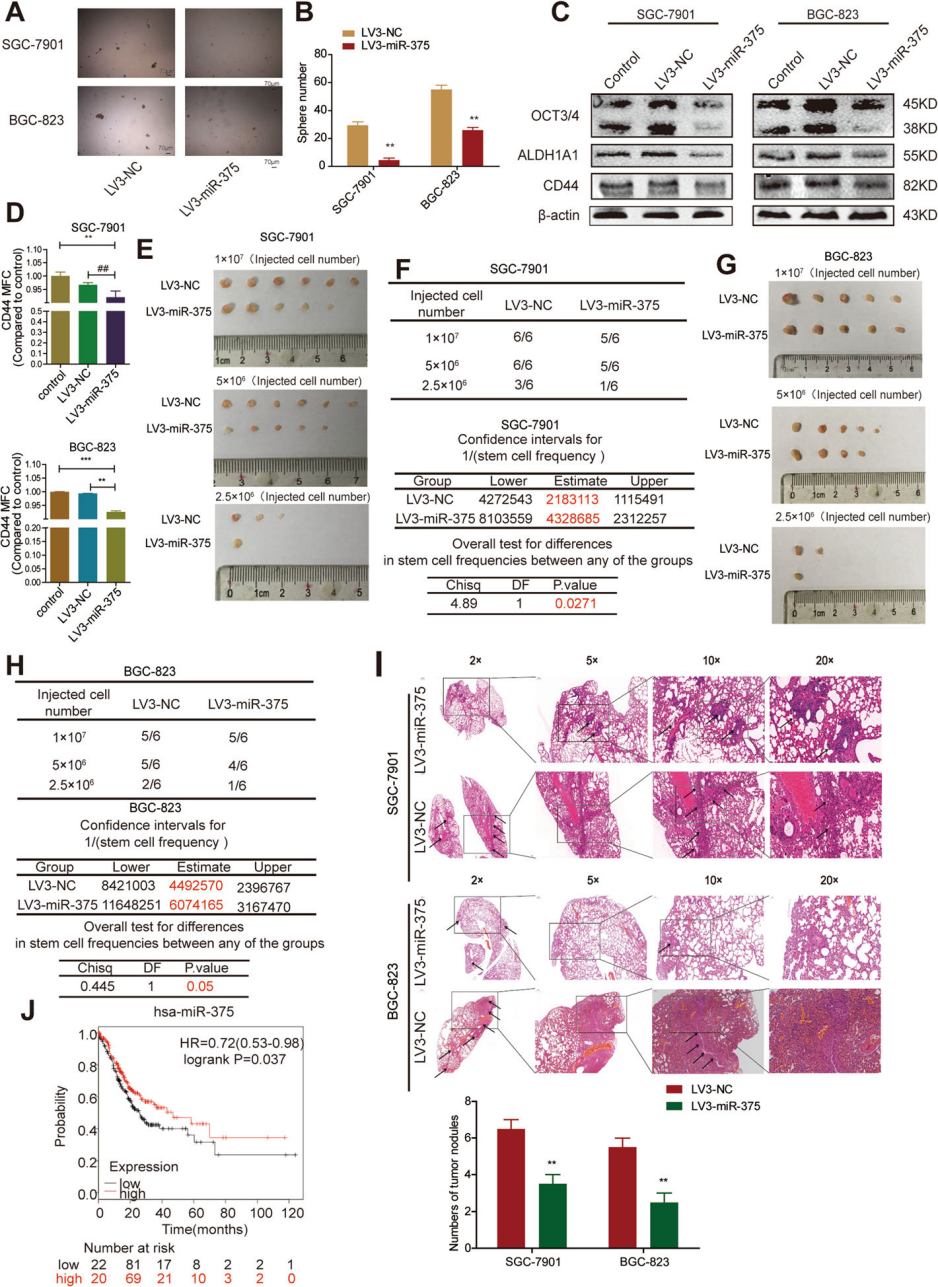
MiR-375 attenuates the stemness of GC cells. **A**, **B** The sphere size and number were examined in GC cells with or without miR-375 overexpression. **C** The protein expression of stemness markers was evaluated in GC cells with or without miR-375 overexpression. **D** CD44+ sub-population was determined in GC cells with or without miR-375 overexpression. **E** The images of tumors derived from different numbers of SGC7901 cells with or without miR-375 overexpression. **F** The tumor formation rate and confidence intervals for 1/(stem cell frequency) were calculated based on the results shown in **E**. **G** The images of tumors derived from different numbers of BGC-823 cells with or without miR-375 overexpression. **H** The tumor formation rate and confidence intervals for 1/(stem cell frequency) were calculated based on the result shown in **G**. **I** HE staining images of lung obtained from mice with injection of GC cells with or without miR-375 overexpression. **J** The correlation between miR-375 expression and the overall survival probability of GC patients was evaluated using the online analysis tool (http://kmplot.com). Data are presented as the mean ± SD, n ≥ 3, **p < 0.01 vs control, ^##^p < 0.01 vs LV3-NC

To explore the roles of miR-375 in GC progression, we constructed two GC cell lines (BGC-823, SGC-7901) with or without miR-375 stable overexpression (LV3-miR-375 vs LV3-NC) by lentivirus infection. The overexpression efficiency was verified by qRT-PCR and fluorescence observation (Supplementary Figure S1A – C). Since we previously showed that miR-375 can inhibit *Helicobacter pylori*-induced gastric carcinogenesis [31] and tumor cell stemness is regarded as one of the main factors contributing to the recurrence and metastasis of tumors, we further investigated the effects of miR-375 on GC cell stemness. Since the non-adherent tumor cell spheres are highly enriched with CSC-like cells compared to the adherent tumor cells [51], we determined whether miR-375 has effects on sphere-formation ability and found that both the size and amount of spheres were decreased in GC cells with miR-375 overexpression (Fig. 1A, B). Surprisingly, SGC-7901 cells overexpressing miR-375 could hardly form spheres. Additionally, the expression of pluripotent transcription factors (OCT3/4, ALDH1A1, CD44) was significantly decreased by miR-375 overexpression (Fig. 1C). Besides, the CD44+ sub-population of GC cells with stemness was decreased by miR-375 overexpression (Fig. 1D). Furthermore, we found that the tumorigenic ability of GC cells with miR-375 overexpression was decreased, which was evident by the decreased tumor formation rate and stem cell frequency using the tumor-limiting dilution assay (Fig. 1E–H). Consistently, GC cells with or without miR-375 overexpression were injected into nude mice through the tail vein. Six weeks later, the metastatic ability of miR-375-overexpressed cells was weaker than that of NC group, as characterized by the decrease of metastatic nodules (Fig. 1I). Notably, the online dataset analysis (Kaplan Meier-plotter tool: http://kmplot.com) showed that GC patients with a higher level of miR-375 held a longer lifetime (Fig. 1J). These results suggest that miR-375 can reduce the stemness of GC cells.

### SLC7A11 is identified as the direct target of miR-375

**Fig. 2:**
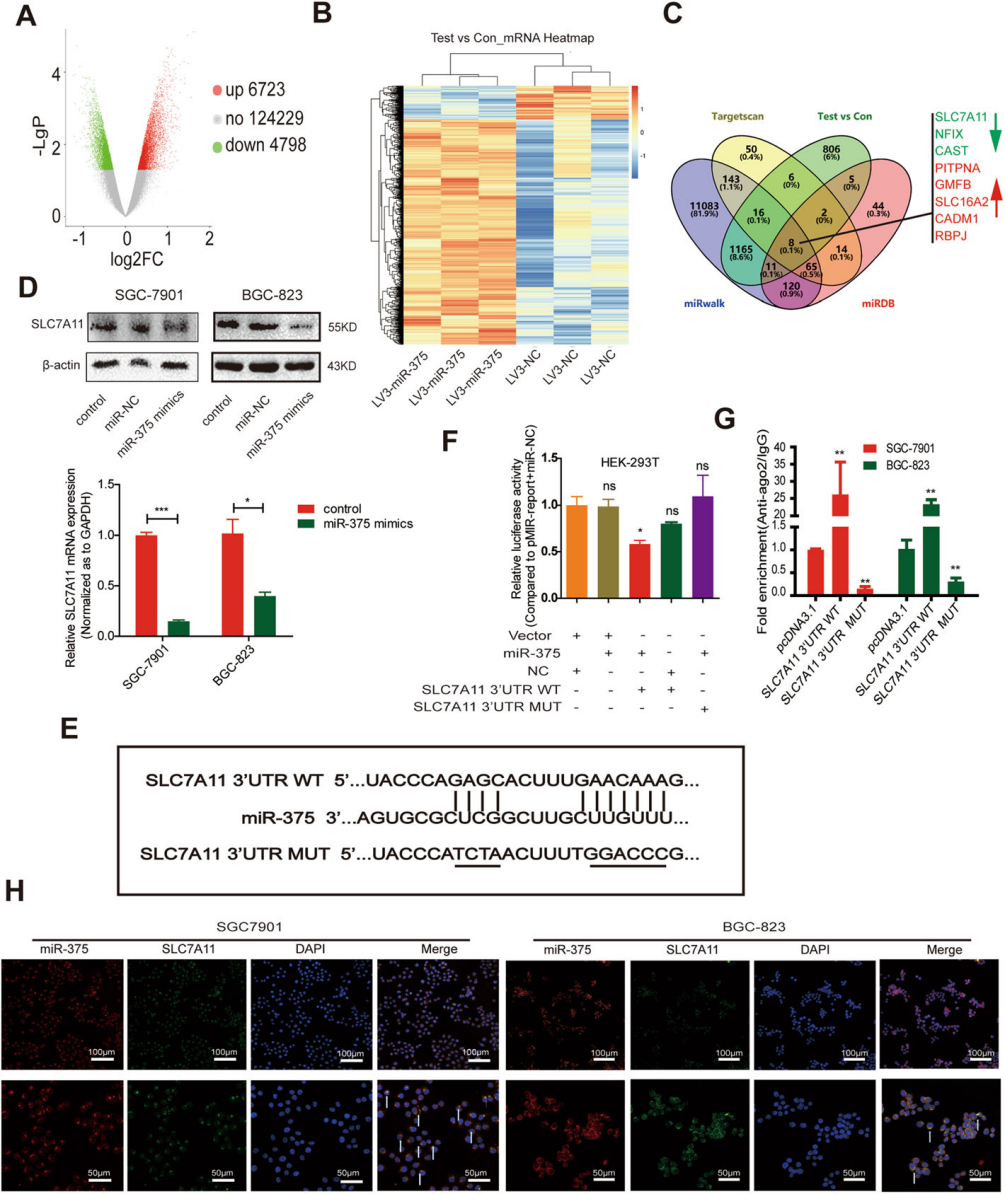
SLC7A11 is a direct target of miR-375 in GC cells. **A** The volcano map indicating the number of mRNAs in SGC-7901 cells with or without miR-375 overexpression. **B** The mRNA heatmap showing the change of mRNA expression in SGC-7901 cells with or without miR-375 overexpression. **C** The venn map showing the potential targets of miR-375. **D** SLC7A11 protein expression is detected in GC cells with or without miR-375 overexpression. Data are presented as the mean ± SD, n ≥ 3, *p < 0.05, **p < 0.001 vs control. **E** The diagram indicating the binding of miR-375 on SLC7A11. **F** Luciferase reporter analysis was performed to determine the effect of miR-375 on the activity of SLC7A11 3′UTR WT and SLC7A11 3′UTR MUT. Data are presented as the mean ± SD, ns means no significance, n ≥ 3, *p < 0.05 vs NC+vector. **G** RIP assay was constructed to measure miR-375 level in RNA pulled down by Anti-Ago2 in GC cells with SLC7A11 3′UTR WT or SLC7A11 3′UTR MUT overexpression. Data are presented as the mean ± SD, n ≥ 3, **p < 0.01 vs pMIR-report empty group. **H** RNA-FISH was performed to evaluate the co-localization of miR-375 and SLC7A11 in GC cells

Then, we set out to explore the mechanisms by which miR-375 inhibits GC cell stemness. We screened out the possible downstream targets of miR-375 through performing gene-chip sequencing analysis. Due to the expression level of miR-375 in SGC-7901 being relatively low among several GC cell lines (Supplementary Figure S1D), we chose SGC-7901 LV3-miR-375 and LV3-NC as the experimental models. It was found that 6723 genes were increased and 4798 genes were decreased in SGC-7901 cells with miR-375 overexpression (Fig. 2A, B), among which SLC7A11, NFIX, CAST, PITPNA, GMFB, SLC16A2, CADM1, and RBPJ were predicted to be the potential targets of miR-375 through online database prediction (Targetscan, miRDB, miRwalk) (Fig. 2C). Since SLC7A11, NFIX, and CAST were decreased by miR-375 overexpression and targeting SLC7A11 has recently been shown to kill colorectal CSCs [52], SLC7A11 was chosen for further research. As expected, SLC7A11 protein and mRNA levels were reduced in GC cells with miR-375 overexpression (Fig. 2D). To further demonstrate that miR-375 can directly target SLC7A11, we constructed luciferase reporter plasmids containing SLC7A11 3′UTR with miR-375 binding site mutant or not (Fig. 2E) and found that the luciferase activity of pMIR-3′UTR-WT was remarkably suppressed by miR-375 overexpression, while no significant effect on the activity of pMIR-3′UTR-MUT was observed (Fig. 2F). Additionally, miR-375 was increasingly enriched in RNA complex pulled down by Ago2 in cells with SLC7A11-3′UTR-WT overexpression but decreased in cells with SLC7A11-3′UTR-MUT overexpression (Fig. 2G). Furthermore, RNA-FISH assays showed a clear overlap between miR-375 and SLC7A11 in two GC cell lines, mainly in the cytoplasm (Fig. 2H). These results indicate that miR-375 can directly target SLC7A11 in GC cells.

### MiR-375 triggers ferroptosis via targeting SLC7A11 in GC cells

**Fig. 3:**
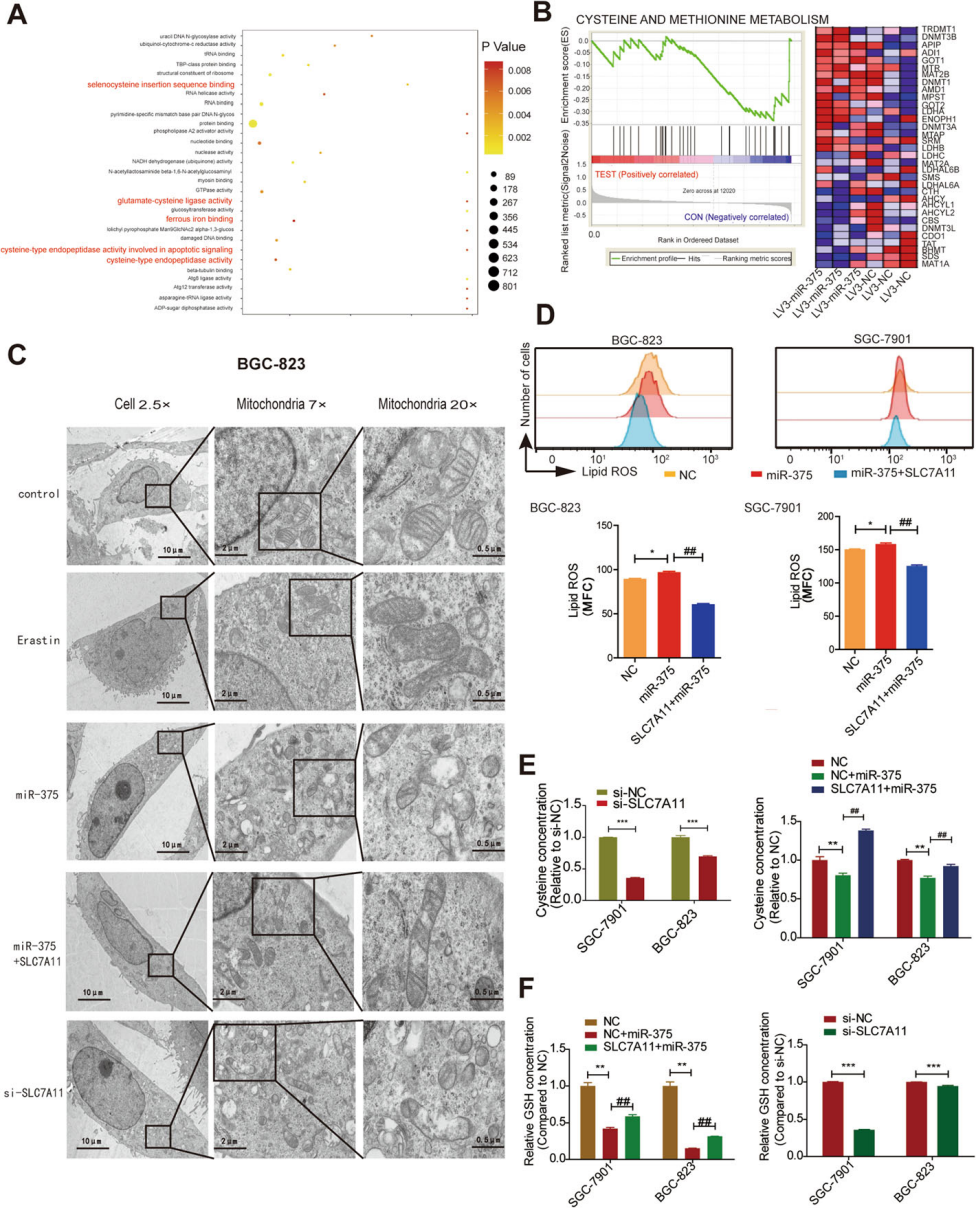
MiR-375 triggers ferroptosis through targeting SLC7A11. **A** KEGG pathway enrichment analysis in SGC-7901 cells with or without miR-375 overexpression. **B** GSEA analysis in SGC-7901 cells with or without miR-375 overexpression. **C** Transmission electron microscopy (TEM) assay on the mitochondria of GC cells with erastin treatment, miR-375 overexpression, SLC7A11 knockdown, or miR-375 overexpression as well as SLC7A11 overexpression. **D** Lipid peroxidation level was measured in GC cells with miR-375 overexpression as well as SLC7A11 overexpression or not. *p < 0.05 vs NC, ^##^p < 0.01 vs miR-375. **E** Cysteine concentration was examined in the cells as depicted in **D**. ***p < 0.001 vs si-NC, ^##^p < 0.01 vs NC+miR-375. **F** GSH concentration was detected in the cells described in **D**. **p < 0.01, ***p < 0.001 vs NC, ^##^p < 0.01 vs NC+miR-375. Data are presented as the mean ± SD, n ≥ 3

Since SLC7A11 is a critical suppressor of ferroptosis through uptaking cystine into the cells, we wondered whether miR-375 can trigger ferroptosis via targeting SLC7A11 in GC cells. Indeed, analysis on our gene-chip results revealed that cysteine metabolism- and ferroptosis-related signaling pathways (*Ferrous iron binding*, *Cysteine-type endopeptidase activity involved in apoptotic signaling*, *Cysteine-type endopeptidase activity*, *Glutamate-cysteine ligase activity*, and *Selenocysteine insertion sequence binding*) were enriched in GC cells with miR-375 overexpression (Fig. 3A). Consistently, GSEA analysis obtained the similar result that *KEGG_CYSTEINE AND METHIONINE METABOLISM* was positively correlated with miR-375 overexpression (Fig. 3B). Then, three independent siRNAs against SLC7A11 were used and we chose SLC7A11-2 for the subsequent experiment as SLC7A11-2 showed the strongest knockdown efficiency (Supplementary Figure S1E–F). Additionally, transmission electron microscopy (TEM) assay showed that the mitochondria became smaller and membrane density was increased with vestigial cristae in GC cells with miR-375 overexpression or SLC7A11 knockdown and miR-375-mediated effect was reversed by SLC7A11 overexpression (Fig. 3C). The efficiency of SLC7A11 overexpression was verified later (Supplementary Figure S1G – I). Erastin, a ferroptosis inducer, served as a positive control. Furthermore, lipid ROS level was increased in cells overexpressing miR-375, which was rescued by SLC7A11 overexpression (Fig. 3D). Cysteine, which is a product of cystine conversion, was detected and it was found that the amount of cysteine was reduced by miR-375 overexpression (Fig. 3E), as well as the synthesis of GSH (Fig. 3F); these effects were attenuated by SLC7A11 overexpression (Fig. 3E, F). Moreover, SLC7A11 knockdown indeed decreased cysteine and GSH levels (Fig. 3E, F). However, it was found that the iron concentration in tumors derived from BGC-823 cells with or without miR-375 overexpression is similar (Supplementary Figure S2). Thus, our results demonstrate that miR-375 can induce ferroptosis in GC cells through targeting SLC7A11, but not altering the cellular iron concentration.

### MiR-375 attenuates the stemness of GC cells dependent on SLC7A11

**Fig. 4:**
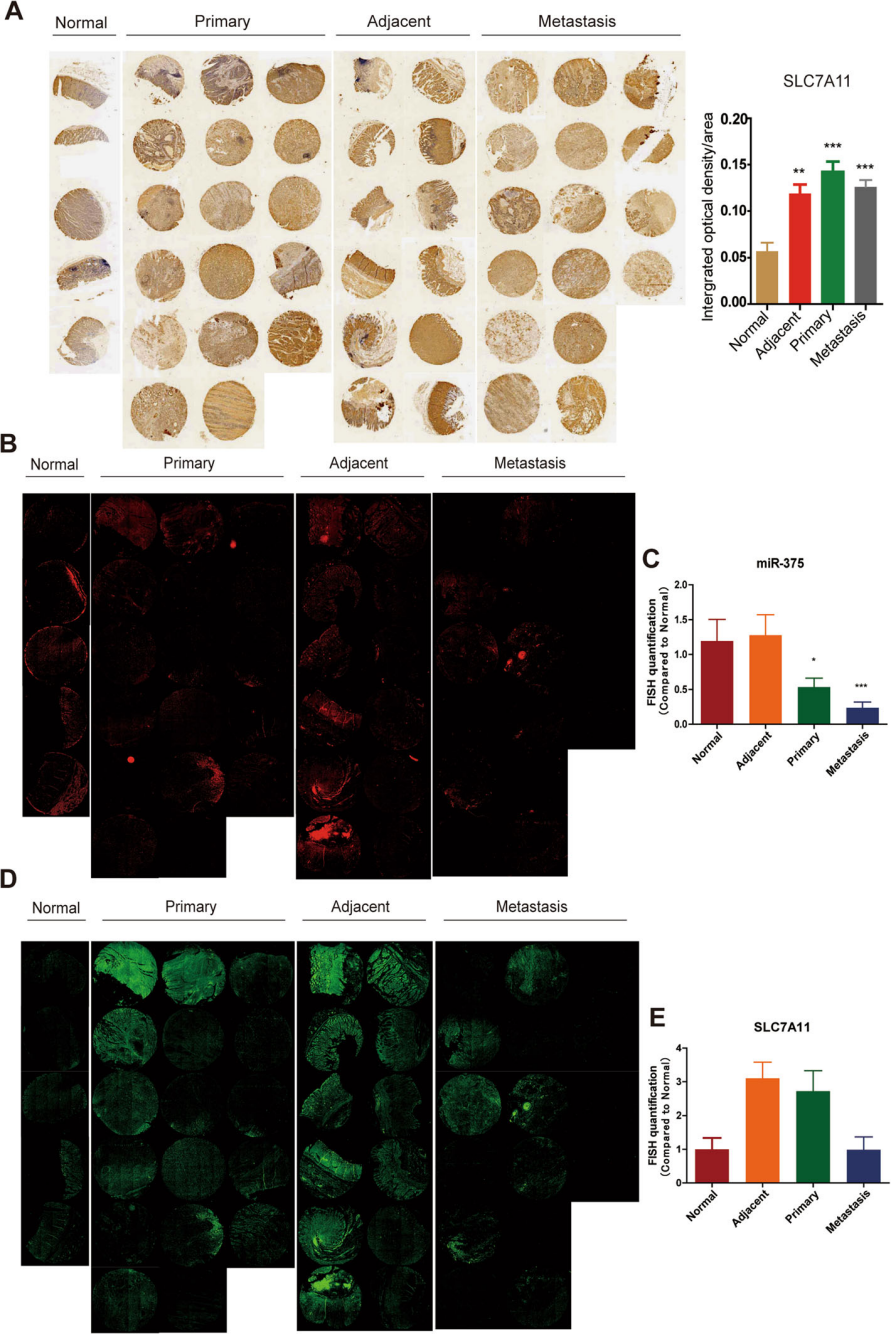
The expression levels of miR-375 and SLC7A11 in tissue chips. **A** Tissue chip containing different types of tissues as indicated was used to detect SLC7A11 protein level by IHC. **p < 0.01, ***p < 0.001 vs normal tissues. **B** Tissue chip was used to detect miR-375 level through RNA-FISH. **C** The fluorescence quantification was calculated based on the result. *p < 0.05, ***p < 0.001 vs normal tissues. **D**, **E** Tissue chip was used to detect SLC7A11 mRNA level through RNA-FISH and fluorescence quantification was shown

**Fig. 5:**
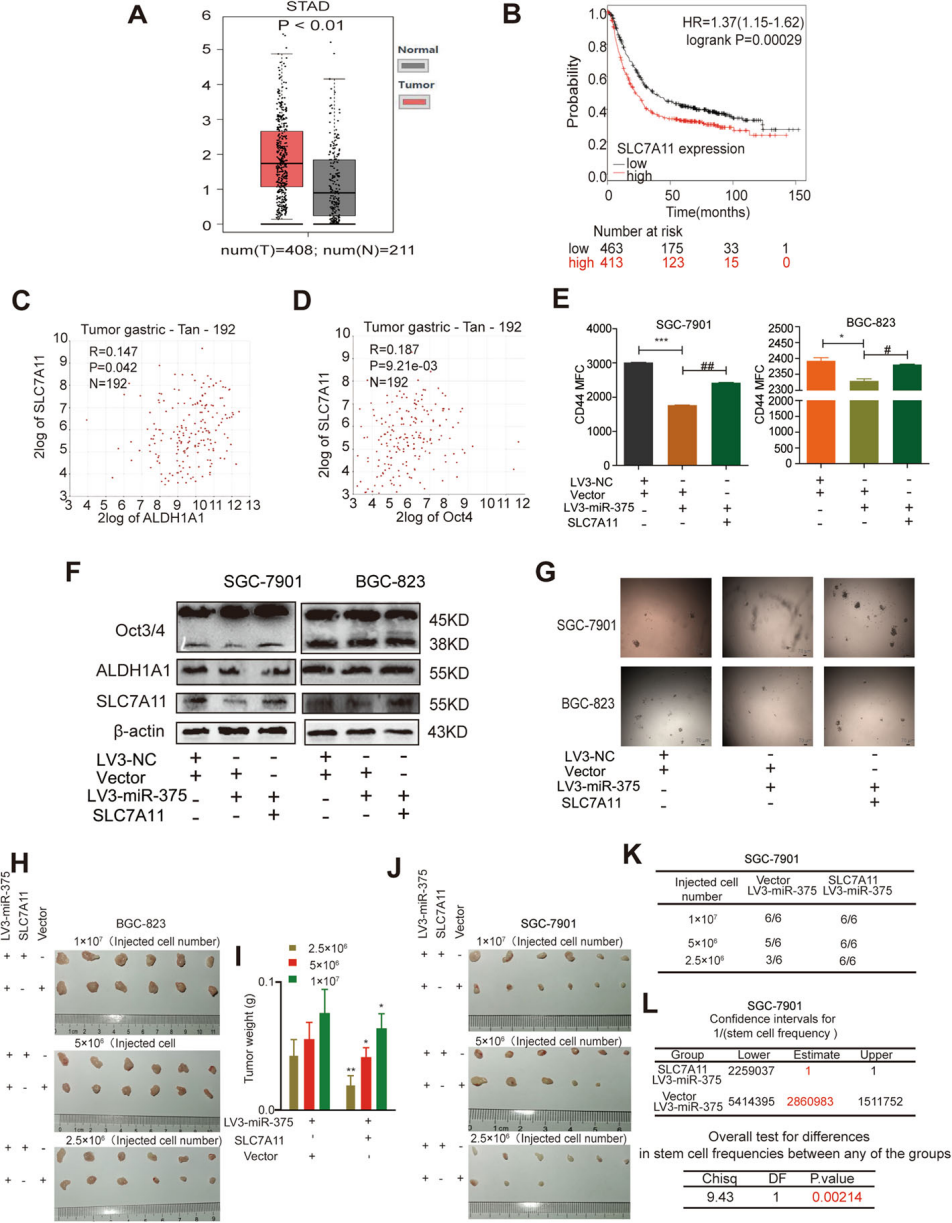
MiR-375 attenuates the stemness of GC cells by targeting SLC7A11. **A** Online dataset (http://gepia.cancer-pku.cn/index.html) was used to detect SLC7A11 mRNA level in GC (T) and normal (N) tissues. **B** The correlation between SLC7A11 mRNA expression and the overall survival probability of GC patients was evaluated using the online analysis tool (http://kmplot.com). **C**, **D** The correlation between SLC7A11 mRNA expression and ALDH1A1 (**C**) or Oct4 (**D**) mRNA expression was evaluated using the online analysis tool (R2: Genomics Analysis and Visualization Platform (https://hgserver1.amc.nl/cgi-bin/r2/main.cgi)). **E** CD44+ sub-population was determined in GC cells with miR-375 overexpression plus SLC7A11 overexpression or not. Data are presented as the mean ± SD, n ≥ 3, *p < 0.05, ***p < 0.001 vs LV3-NC+Vector, ^#^p < 0.05, ^##^p < 0.01 vs Vector+LV3-miR-375. **F** The protein expression of stemness markers was examined in the GC cells. **G** The sphere size and number were evaluated in the GC cells. **H** Tumor images derived from BGC-823 cells described in **H**. **I** Tumor weight of tumors shown in **I**. **J** Tumor images derived from SCG7901 cells described in **J**. **K**, **L** Tumor formation rate (**K**) and confidence intervals for 1/(stem cell frequency) (**L**) were calculated based on the result shown in **L**. Data are presented as the mean ± SD, n ≥ 3, *p < 0.05, **p < 0.01 vs NC

We further examined SLC7A11 expression through tissue chip containing normal gastric epithelial tissues, GC tissues with or without metastasis and found that SLC7A11 protein was highly expressed in GC tissues compared to that in normal gastric epithelial tissues (Fig. 4A). The expression level of miR-375 in normal gastric epithelial tissues was higher than that in GC tissues, while the SLC7A11 mRNA level was significantly lower (Fig. 4B–E). The results appeared an opposite relationship between miR-375 and SLC7A11. GEPIA database (http://gepia.cancer-pku.cn/index.html) also showed a consistent result that SLC7A11 exhibited a higher level in GC tissues than that in normal tissues (Fig. 5A). Online dataset showed that SLC7A11 expression is negatively correlated with the survival probability of GC patients (http://kmplot.com) (Fig. 5B) and positively correlated with stemness marker (ALDH1A1, Oct4) expression (R2: Genomics Analysis and Visualization Platform (http://r2.amc.nl)) (Fig. 5C, D). These results polished us to explore whether miR-375 attenuates the stemness of GC cells through targeting SLC7A11. As shown in Fig. 5E, miR-375 overexpression-induced downregulation of CD44^+^ sub-population with stemness was attenuated by SLC7A11 overexpression. Furthermore, the decreased expression of stemness markers led by miR-375 overexpression was partially reversed by SLC7A11 overexpression (Fig. 5F). Moreover, the reduced spheroid formation ability resulted by miR-375 overexpression was rescued by SLC7A11 overexpression (Fig. 5G). Notably, although all BGC-823 cell lines could form tumors, cells with miR-375 overexpression as well as SLC7A11 overexpression showed an increase of tumor size and weight compared with that in cells with miR-375 overexpression alone (Fig. 5H, I). Moreover, the decreased tumorigenic ability of SGC-7901 cells led by miR-375 overexpression was rescued by SLC7A11 overexpression, which was evident by the increased tumor formation rate and stem cell frequency (Fig. 5J–L).

**Fig. 6:**
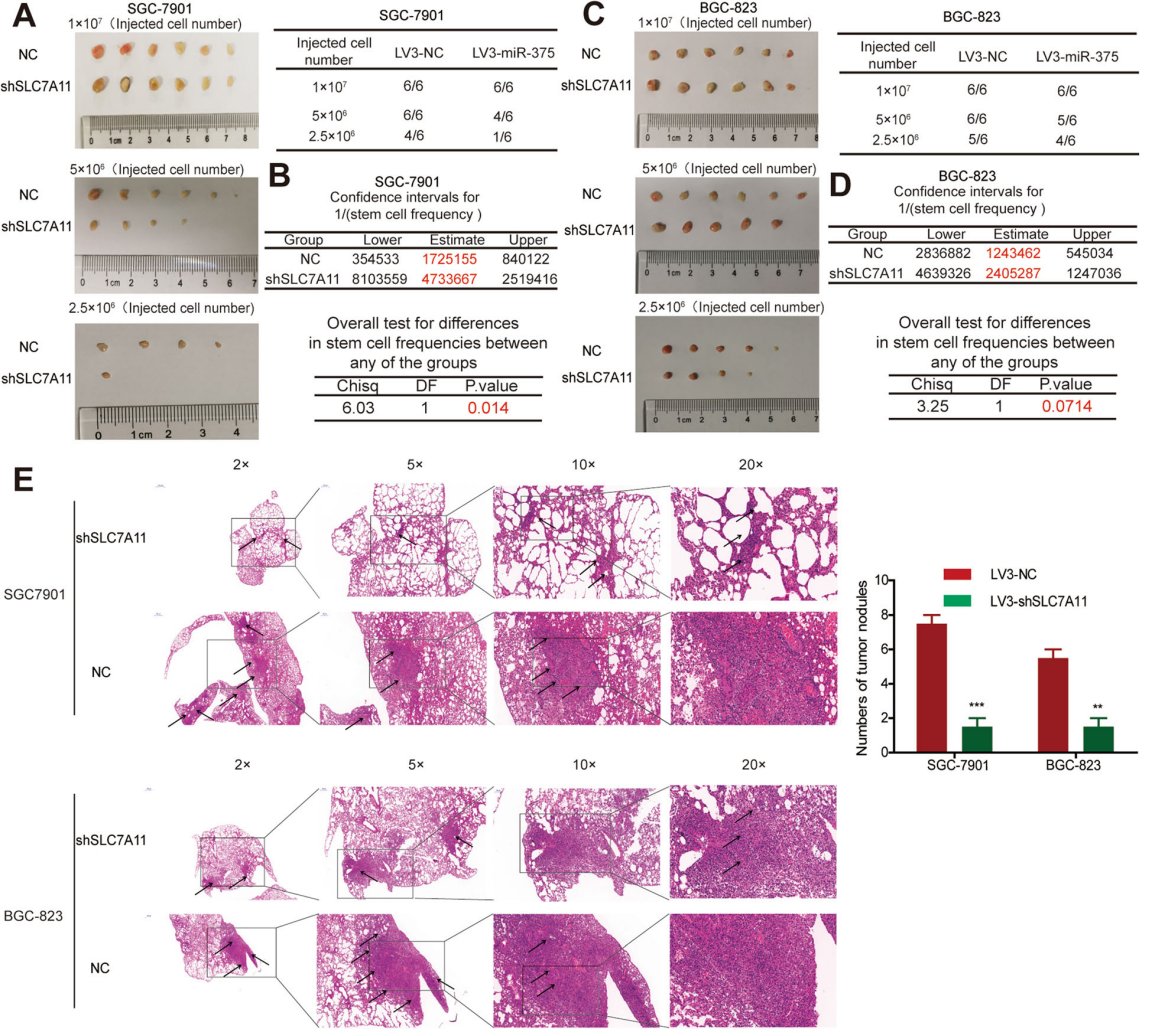
Knockdown of SLC7A11 suppresses the tumor-initiating and metastatic ability of GC cells. **A** Tumor images and formation rate derived from SGC7901 cells with or without SLC7A11 knockdown. **B** The confidence intervals for 1/(stem cell frequency) was calculated based on the results shown in **A**. **C** Tumor images and formation rate derived from BGC823 cells with or without SLC7A11 knockdown. **D** The confidence intervals for 1/(stem cell frequency) was calculated based on the results shown in **C**. **E** HE staining images of lung obtained from mice with injection of GC cells with or without miR-375 overexpression

Further in vivo experiments revealed that SLC7A11 knockdown indeed suppressed the tumor-initiating ability (Fig. 6A, C), stem cell frequency of GC cells (Fig. 6B, D), and metastatic capacity (Fig. 6E). Additionally, the ferroptosis inducer (erastin, sorafenib, sulfasalazine) indeed attenuated the stemness of GC cells, which was rescued by SLC7A11 overexpression (Supplementary Figure S3). Taken together, these results indicate that miR-375 suppresses the stemness of GC cells dependent on SLC7A11.

### MiR-375 attenuates the stemness of GC cells mainly through triggering ferroptosis

**Fig. 7:**
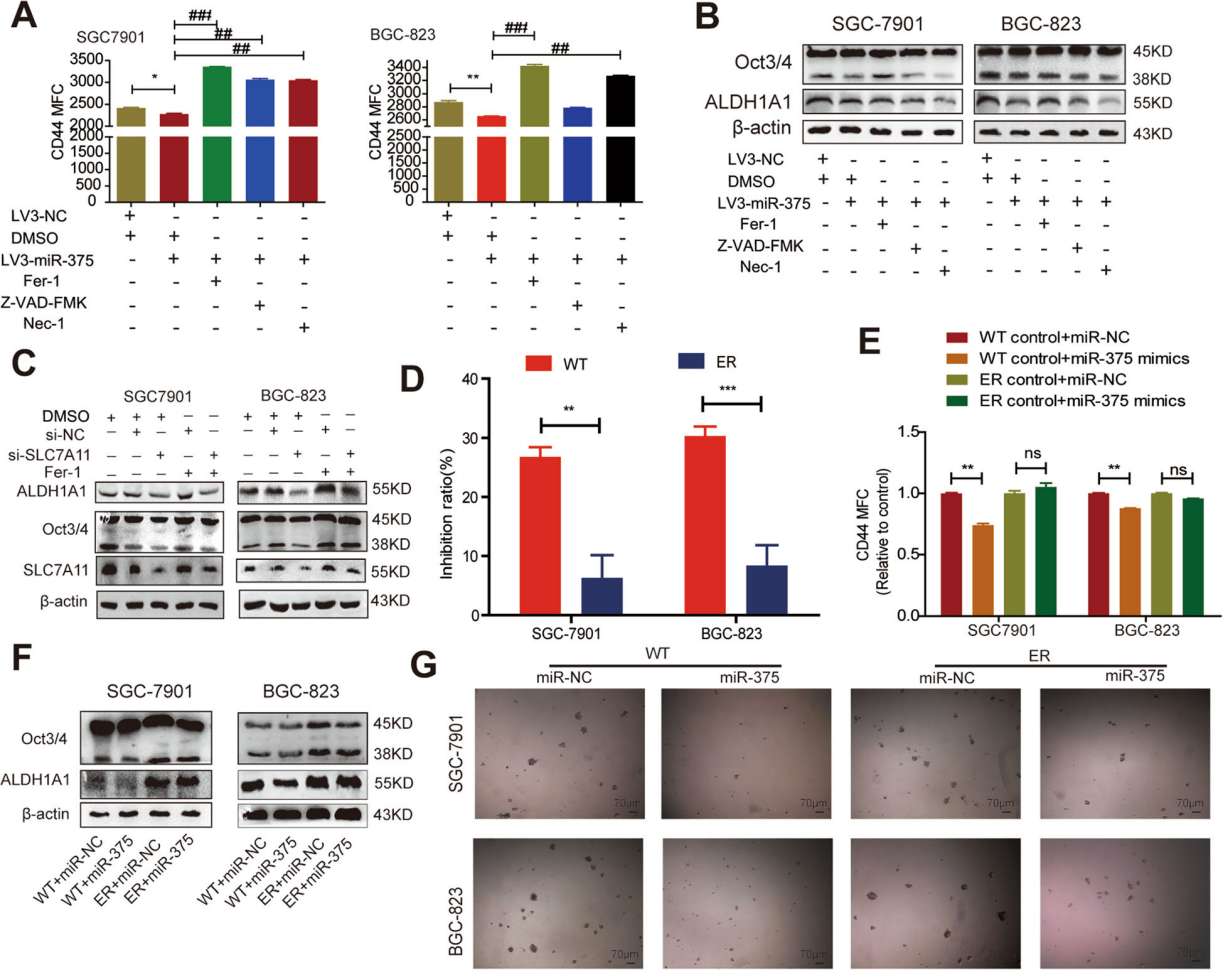
MiR-375 attenuates the stemness of GC cells mainly through inducing ferroptosis. **A** CD44+ sub-population was examined in GC cells with miR-375 overexpression plus Fer-1, Z-VAD-FMK, or Nec-1 treatment. Data are presented as the mean ± SD, n ≥ 3, *p < 0.05, **p < 0.01 vs LV3-NC+DMSO; ^##^p < 0.01, ^###^p < 0.001 vs LV3-miR-375+DMSO. **B** The expression of stemness markers was detected in the cells depicted in **A**. **C** The expression of stemness markers was determined in GC cells with SLC7A11 knockdown plus Fer-1 treatment or not. **D** The inhibition ratio of erastin was measured in WT and ER cells. Data are presented as the mean ±ean ar ≥ 3, **p < 0.01, ***p < 0.001 vs WT or ER. **E** CD44+ sub-population was assessed in WT and ER cells with miR-375 overexpression or miR-NC. Data are presented as the mean ±re pre ≥ 3, **p < 0.01 vs control+miR-NC; ns, no significance. **F** The expression of stemness markers was examined in the cells described in **E**. **G** Sphere-formation ability was evaluated in the cells depicted in **E**

Then, we investigated whether the miR-375/SLC7A11 axis regulates the stemness of GC cells through triggering ferroptosis. Considering that miR-375/SLC7A11 axis may play a role through other forms of cell death instead of inducing ferroptosis, GC cells with miR-375 overexpression or SLC7A11 knockdown were treated with Fer-1 (10 μM), apoptosis inhibitor Z-VAD-FMK (20 μM) [53], or necrosis inhibitor Nec-1 (0.1 mM) [54, 55], and following by examining the stemness of GC cells. As shown in Fig. 7A, the decreased CD44+ sub-population of GC cells induced by miR-375 overexpression was rescued by Fer-1 treatment, Z-VAD-FMK treatment, and Nec-1 treatment in SGC7901 and BGC-823 cells, among which Fer-1 treatment exhibited the strongest effect. A similar effect was observed upon detecting the expression of stemness markers (Fig. 7B). Consistently, the decreased expression of stemness markers caused by SLC7A11 knockdown can be significantly reversed by Fer-1 (Fig. 7C). Additionally, we constructed erastin-resistant (ER) GC cells and the resistance index was confirmed (Fig. 7D). It was found that ER GC cells exhibited a stronger stemness than the parental GC cells, which was evident by the increase of CD44+ sub-population (Fig. 7E), stemness marker expression (Fig. 7F), and sphere-formation ability (Fig. 7G). Notably, we found that miR-375 overexpression had little effect on the stemness of ER GC cells (Fig. 7E–G). Taken together, although we did not rule out the role of apoptosis and necrosis in miR-375-induced effects, our results demonstrate that miR-375 suppresses the stemness of GC cells mainly through triggering ferroptosis.

## Discussion

**Fig. 8:**
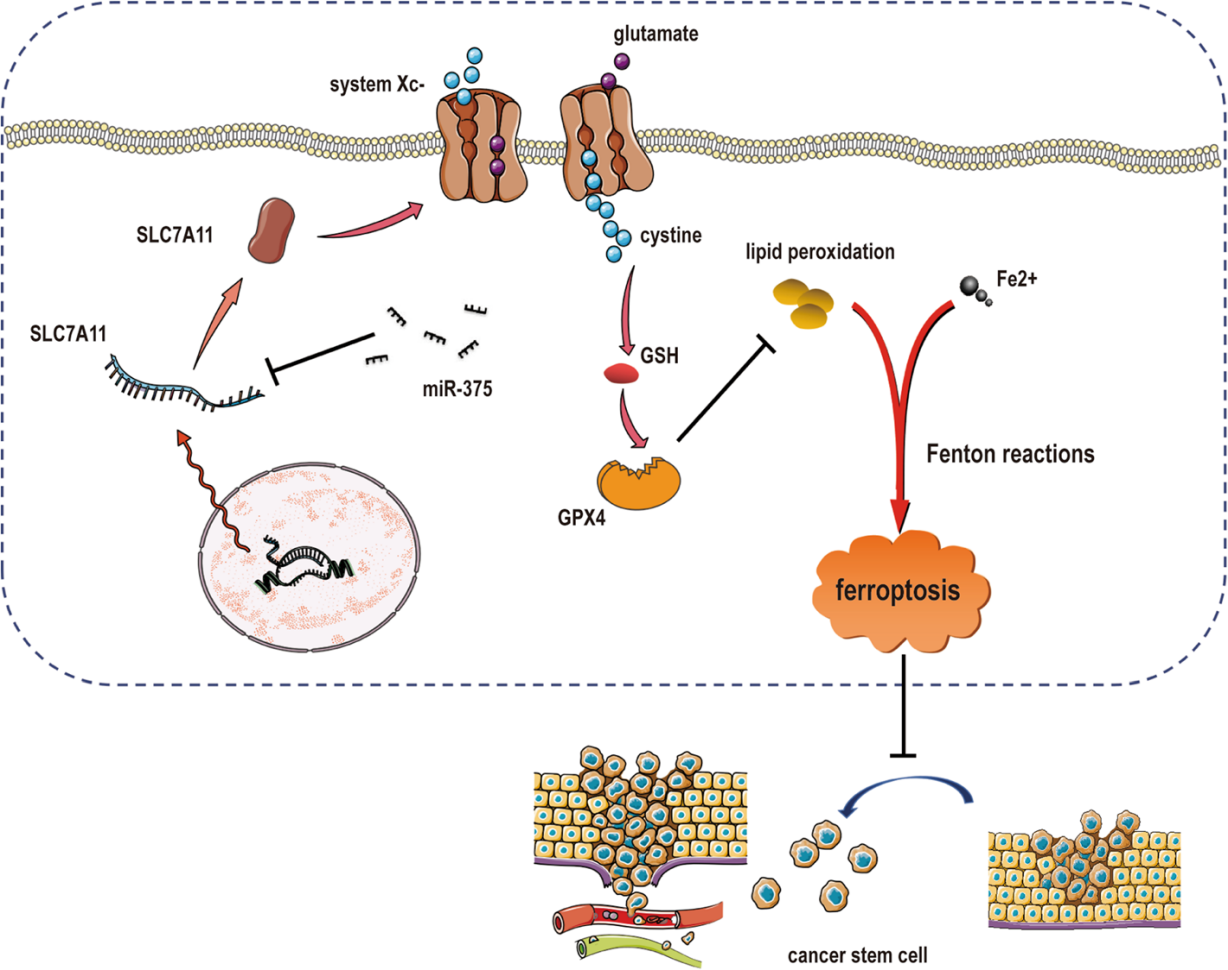
Proposed model that miR-375 suppresses the stemness of GC cells. SLC7A11 is selective for uptaking cystine into the cell in exchange for glutamate. This system synthesizes glutathione (GSH) by taking extracellular cystine into the cell and thus represses lipid peroxidation in ferroptosis; this process is suppressed by miR-375 which can directly target SLC7A11. Additionally, miR-375 has no effect on iron concentration

Our and other previous studies have shown that miR-375 is lowly expressed in GC [31, 56, 57]. And miR-375 can inhibit tumor growth, metastasis and invasion by suppressing the EMT (epithelial-mesenchymal transition) process in various cancers [58–60], including GC [61]. Since the tumor progression has been attributed to the existence of CSCs [62] and we recently showed that miR-375 can attenuate the stemness of breast cancer cells [32], we assumed that miR-375 could regulate the stemness of GC cells too. In the present study, we discovered that miR-375 indeed reduced the stemness of GC cells, which was evident by the decrease of cell sub-population with stemness, stemness marker expression, sphere-formation, and tumor-initiating ability. Combined with the microarray analysis, we further revealed that miR-375 exerts its effects through targeting SLC7A11 and thus triggering ferroptosis (Fig. 8). To our knowledge, this is the first study showing the effect of miR-375 on the stemness of GC cells.

SLC7A11 is one of subunit cystine/glutamate transporter which plays an important role in cell metabolism [18]. Importantly, SLC7A11 is the critical suppressor of ferroptosis through increasing the uptake of cystine and thus maintaining glutathione synthesis [63] and targeting SLC7A11 could specifically kill colorectal CSCs [52]. Indeed, the cysteine metabolism ferroptosis-related signaling pathways were enriched in GC cells with miR-375 overexpression (Fig. 3A). Our recent work indicates that miR-375 suppresses the stemness of breast cancer cells through targeting JAK2 and thus inhibiting the JAK2/STAT3 pathway [32], although we did not exclude that miR-375 might attenuate the stemness of GC cells through targeting the JAK2/STAT3 pathway in this work, we suggest that miR-375 could regulate the stemness of tumor cells through different mechanisms in different tumors. Additionally, the previous study has shown that the miR-375/SLC7A11 axis suppresses oral squamous cell carcinoma proliferation and invasion [64]; this is consistent with our work. We speculate that the miR-375/SLC7A11 axis might be a common phenomenon in tumor progression, which should be explored in the future. Furthermore, through tissue microarray analysis, it was found that SLC7A11 was highly expressed in GC tissues compared to normal gastric epithelial tissues. As expected, miR-375 was lowly expressed in GC tissues compared to normal gastric epithelial tissues, which exhibited a negative correlation with SLC7A11 expression. However, the expression of SLC7A11 in GC tissues with or without metastasis exhibits a similar protein level, which seems to present a contradictory result; that is, SLC7A11 is not positively correlated with metastasis; this might be due to the limited tissue samples. Furthermore, the EMT- and stemness-related traits exhibit a dynamics of phenotypic heterogeneity during progression [65], and CSCs have a metabolic heterogeneity and flexibility through reprograming their metabolism to flexibly respond to environmental changes [66]. Thus, we think that SLC7A11 expression in this limited tissue samples might not reflect the correlation between SLC7A11 expression and stemness or metastasis. Moreover, although the recent studies have shown that miR-375 regulates keratinocyte apoptosis by targeting XIAP [67] and suppresses tumorigenesis and chemoresistance by targeting YAP1 and SP [68], the levels of these target genes XIAP, YAP1, and SP1 were not changed in our microarray analysis. Therefore, we think that miR-375 might function differently on different characteristics of cancers. Notably, as SLC7A11 overexpression just rescued the inhibition of miR-375 on GC cell stemness, in which other target genes might be involved, such as NFIX, which was also downregulated in our microarray analysis, has been shown to regulate murine hematopoietic stem and progenitor cell survival [69] and neural stem cell quiescence [70]. Further studies should be constructed to explore the effects of NFIX on tumor cell stemness and especially validate whether NFIX is involved in miR-375-mediated effects on GC cell stemness.

As we reviewed in the recent work that ferroptosis might be a potential target for killing CSCs [14], here, different ferroptosis inducers (erastin, sorafenib, sulfasalazine) indeed suppressed the stemness of GC cells (Supplementary Figure S3), which was rescued by SLC7A11 overexpression. Additionally, erastin has been shown to specifically kill colorectal CSCs and enhance the chemotherapeutic effect [52]. Notably, the previous studies have shown that triggering ferroptosis through another pathway, that is sequestering iron in lysosomes, also can specifically kill CSCs [71, 72], this is confirmed by a recent study showing that itraconazole can sequester iron in lysosome and thus trigger ferroptosis, which is essential for itraconazole-mediated attenuation on nasopharyngeal carcinoma spheroid stemness [73]. These results suggest that SLC7A11 might be a potential target for killing CSCs and these above ferroptosis inducers could be used for GC treatments, especially the chemo-resistant tumor types, although in vivo experiments are needed. Furthermore, our results indicate that ectopic expression of miR-375 did not alter the iron concentration in GC tumors, which means that miR-375 triggers ferroptosis through targeting SLC7A11 without affecting iron balance in GC. And further studies can be constructed to evaluate the effects of miR-375 on the chemotherapy in GC and other targets which might be involved in miR-375-mediated effects on GC cell stemness, such as JAK2 that is confirmed by our previous studies [31, 32]. Moreover, multiple non-apoptosis treatment on GC stemness needs further study; for example, shortened telomere is a hallmark of stem cell senescence and telomere is an important part in stem cell aging and apoptosis [74], and targeting telomeres of CSCs may become a promising treatment against GC without affecting normal cells [75]; this should be explored in the future.

In conclusion, our results demonstrate that miR-375 is a critical suppressor for GC cell stemness by directly targeting SLC7A11. This work suggests that targeting this miR-375/SLC7A11 axis might kill gastric CSCs and thus facilitate the development of GC treatment.

## Supplementary Information

**Additional file 1: Figure S1**. Validation of infection and transfection efficiency. (A and C) The overexpression efficiency was confirmed through analyzing the GFP density and qRT-PCR assay. (D) MiR-375 level was detected in different types of GC cell lines. (E and F) SLC7A11 protein level was determined in GC cells with the transfection of siRNAs against SLC7A11. (G and H) The SLC7A11 mRNA overexpression efficiency was confirmed by qRT-PCR assay after the transfection of SLC7A11 plasmid and vector in GC cells. (I) SLC7A11 protein level was determined in GC cells with the transfection of SLC7A11 plasmid and vector. Data are presented as the mean ± SD, n≥3, *p < 0.05, **p < 0.01, ***p < 0.001 vs control.

**Additional file 2: Figure S2**. Prussian blue staining in tumor derived from GC cells with or without miR-375 overexpression.

**Additional file 3: Figure S3**. SLC7A11 rescues the Ferroptosis inducers-mediated inhibition on GC cell stemness. (A) CD44+ sub-population was detected in GC cells with SAS treatment plus SLC7A11 overexpression. Data are presented as the mean ± SD, n≥3, *p < 0.05, **p < 0.01 vs NC, #p < 0.05, ##p < 0.01 vs NC+SAS. (B) Sphere formation ability was evaluated in GC cells with erastin treatment plus SLC7A11 overexpression. (C) The protein expression of stemness markers was examined in GC cells with ferroptosis inducers (Erastin, SAS, Sorafenib) plus SLC7A11 overexpression.

## Data Availability

All data and materials generated or analyzed for the research are included in this article.

## Abbreviations

GC: Gastric cancer
CSC: Cancer stem cell
ROS: Reactive oxygen species
GSH: Glutathione
Cys: Cysteine
Fer-1: Ferrostatin-1
SAS: Sulfasalazine
Nec-1: Necrostatin-1
LV3 lentiviral vector: pGLV3/H1/GFP+Puro lentiviral vector
LV3-miR-375: miR-375 overexpression lentivirus
LV3-NC: NC lentivirus
LV3-SLC7A11: SLC7A11 overexpression lentivirus
LV3-shSLC7A11: SLC7A11 shRNA lentivirus
LV3-miR-375-SLC7A11: Infected with SLC7A11 overexpression lentivirus and SLC7A11 shRNA lentivirus
RIP: RNA immunoprecipitation
RNA-FISH: RNA-Fluorescence in situ hybridization
CCK-8: Cell Counting Kit-8
GSEA: Gene Set Enrichment Analysis
ER: Erastin-resistant
