# Supplementary figures and images for: MiR-375 reduces the stemness of gastric cancer cells through triggering ferroptosis
Source: Stem Cell Res Ther. 2021 Jun 5;12:325. doi: 10.1186/s13287-021-02394-7 (PMC8180146; doi:10.1186/s13287-021-02394-7)

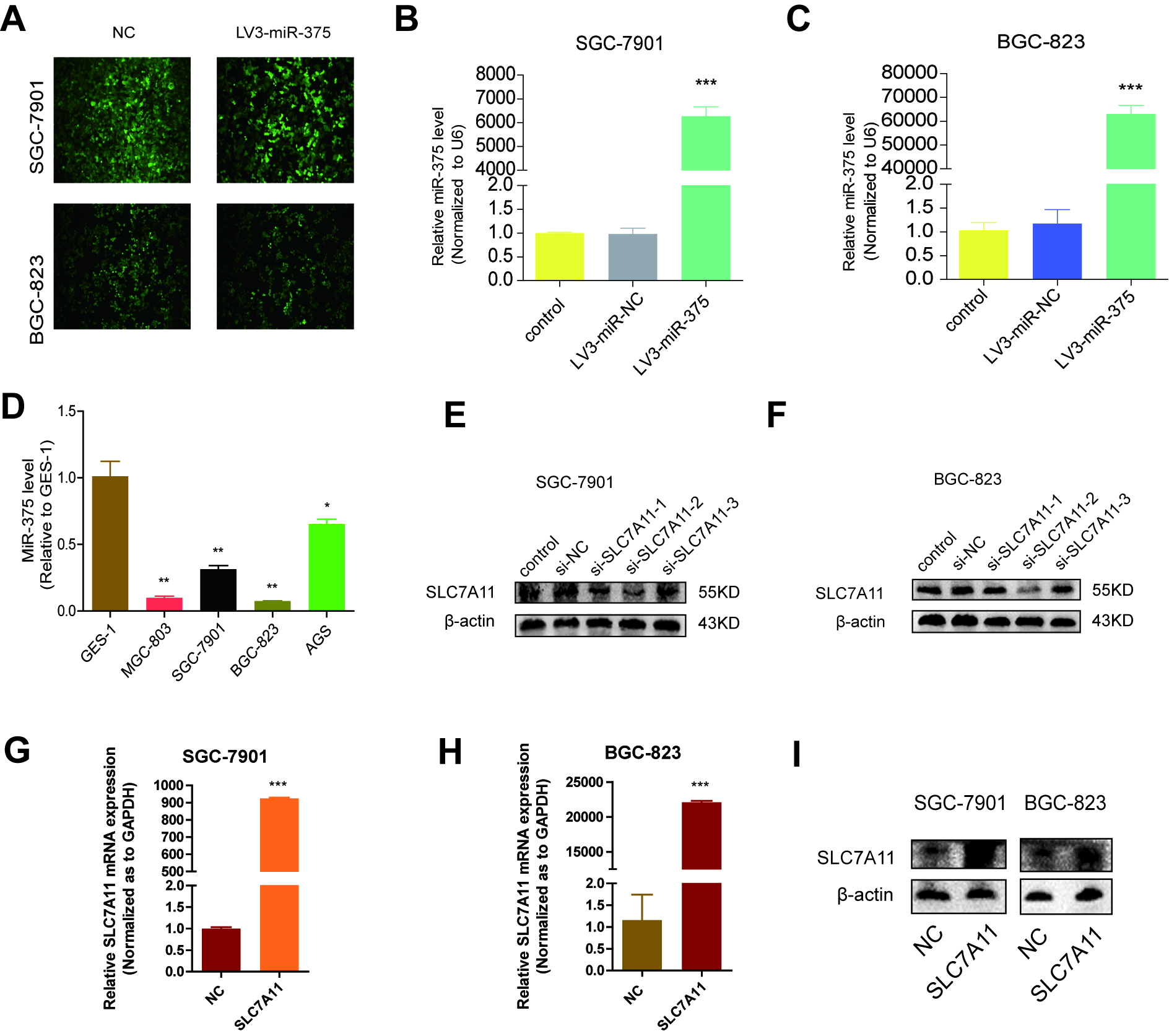

Supplement: Supplementary file 1 — Additional file 1: Figure S1. Validation of infection and transfection efficiency. (A and C) The overexpression efficiency was confirmed through analyzing the GFP density and qRT-PCR assay. (D) MiR-375 level was detected in different types of GC cell lines. (E and F) SLC7A11 protein level was determined in GC cells with the transfection of siRNAs against SLC7A11. (G and H) The SLC7A11 mRNA overexpression efficiency was confirmed by qRT-PCR assay after the transfection of SLC7A11 plasmid and vector in GC cells. (I) SLC7A11 protein level was determined in GC cells with the transfection of SLC7A11 plasmid and vector. Data are presented as the mean ± SD, n≥3, *p < 0.05, **p < 0.01, ***p < 0.001 vs control. [file 13287_2021_2394_MOESM1_ESM.tif]

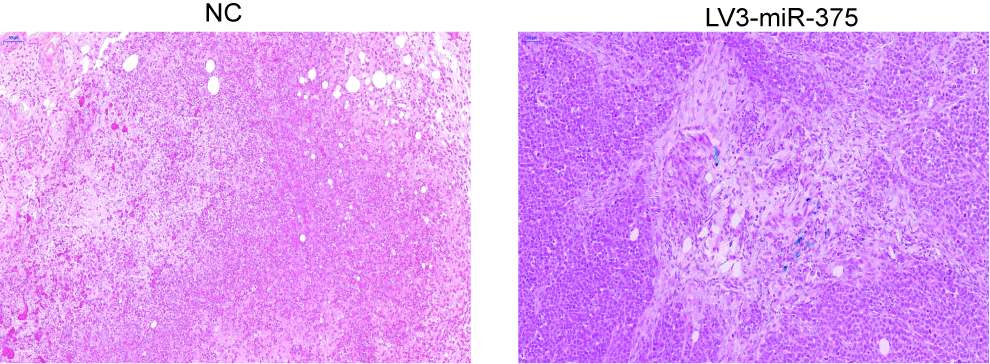

Supplement: Supplementary file 2 — Additional file 2: Figure S2. Prussian blue staining in tumor derived from GC cells with or without miR-375 overexpression. [file 13287_2021_2394_MOESM2_ESM.tif]

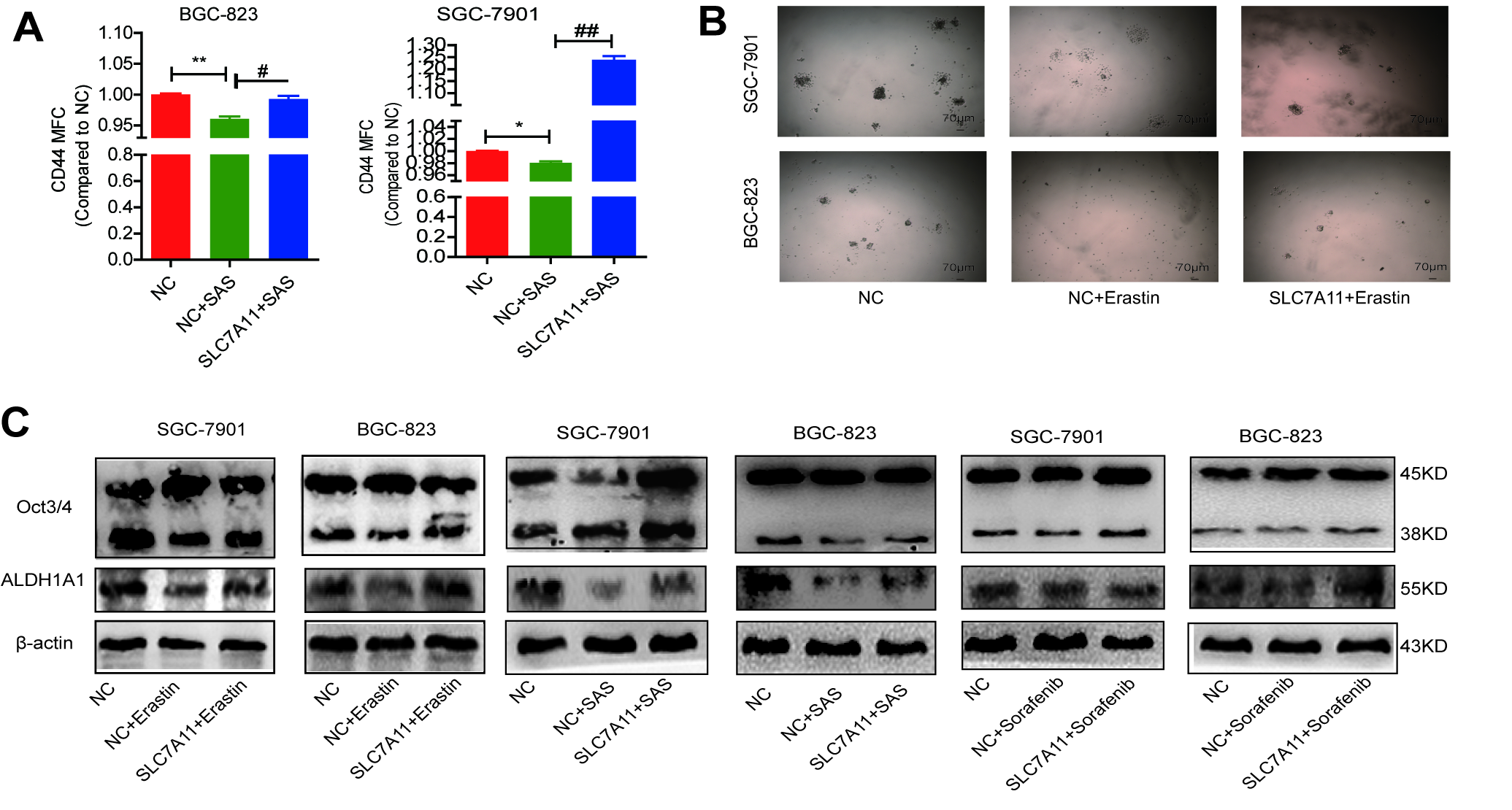

Supplement: Supplementary file 3 — Additional file 3: Figure S3. SLC7A11 rescues the Ferroptosis inducers-mediated inhibition on GC cell stemness. (A) CD44+ sub-population was detected in GC cells with SAS treatment plus SLC7A11 overexpression. Data are presented as the mean ± SD, n≥3, *p < 0.05, **p < 0.01 vs NC, #p < 0.05, ##p < 0.01 vs NC+SAS. (B) Sphere formation ability was evaluated in GC cells with erastin treatment plus SLC7A11 overexpression. (C) The protein expression of stemness markers was examined in GC cells with ferroptosis inducers (Erastin, SAS, Sorafenib) plus SLC7A11 overexpression. [file 13287_2021_2394_MOESM3_ESM.tif]
